# Genomic data of two *Bacillus* and two *Pseudomonas* strains isolated from the acid mine drainage site at Mamut Copper Mine, Ranau, Malaysia

**DOI:** 10.1016/j.dib.2020.106486

**Published:** 2020-11-02

**Authors:** Yi Yik Low, Grace Joy Wei Lie Chin, Collin G. Joseph, Baba Musta, Kenneth Francis Rodrigues

**Affiliations:** a Biotechnology Research Institute, Universiti Malaysia Sabah, Malaysia; b Faculty of Science and Natural Resources, Universiti Malaysia Sabah, Malaysia

**Keywords:** Acid mine drainage (AMD), *Bacillus*, *Pseudomonas*, Heavy metal tolerance

## Abstract

The genomic data of four bacteria strains isolated from the abandoned Mamut Copper Mine, an Acid Mine Drainage (AMD) site is presented in this report. Two of these strains belong to the genus *Bacillus*, while the other two belong to the genus *Pseudomonas.* The draft genome size of *Pseudomonas* sp. strain MCMY3 was 6,396,595 bp (GC: 63.3%), *Bacillus* sp. strain MCMY6 was 6,815,573 bp (GC: 35.2%), *Bacillus* sp. strain MCMY13 was 5,559,059 bp (GC: 35.5%) and *Pseudomonas* sp. strain MCMY15 was 7,381,777 bp (GC: 64.8%). These four genomes contained 493, 495, 495 and 579 annotated subsystems, respectively. The sequence data are available at GenBank sequence read archive with accessions numbers SRX7859406, SRX7859404, SRX7859405 and SRX7293032 for strains MCMY3, MCMY6, MCMY13 and MCMY15, respectively.

## Specifications Table

**Table:** 

Subject	Genetics, Genomics and Molecular Biology
Specific subject area	Raw genomes data of strains MCMY3, MCMY6, MCMY13 and MCMY15 isolated from the acid mine drainage site at Mamut Copper Mine, Ranau, Sabah, Malaysia.
Type of data	Raw genome data
How data were acquired	Sequencer: Illumina HiSeq. 2500 sequencing system
Data format	Raw genome data format: FASTQ files
Parameters for data collection	Sequencing read length and data output Read type: PE150 Output: 140 million average reads, 300 bases, 42 Gb.
Description of data collection	Total genomic (gDNA) were extracted from axenic bacterial cultures with the QIAGEN DNeasy Blood and Tissue kit according to the manufacturer's protocol, library preparation was done using the NEB Next Ultra DNA library Kit (illumina), sequencing was carried out with the Illumina HiSeq 2500 system.
Data source location	Soil samples collected from the Acid Mine Drainage site at Ranau, Malaysia. *Bacillus* sp. MCMY6 strain – 6.030324°; 116.657833° *Bacillus* sp. MCMY13 strain – 6.030845°; 116.661892° *Pseudomonas* sp. MCMY3 strain – 6.030324°; 116.657833° *Pseudomonas* sp. MCMY15 strain – 6.030324°; 116.657833°
Data accessibility	Public repository Repository name: NCBI Sequence Read Archive Data identification number: *Bacillus* sp. MCMY6 strain – SRX7859404 *Bacillus* sp. MCMY13 strain – SRX7859405 *Pseudomonas* sp. MCMY3 strain – SRX7859406 *Pseudomonas* sp. MCMY15 strain – SRX7293032 Direct URL to data: https://www.ncbi.nlm.nih.gov/sra/SRX7859404 https://www.ncbi.nlm.nih.gov/sra/SRX7859405 https://www.ncbi.nlm.nih.gov/sra/SRX7859406 https://www.ncbi.nlm.nih.gov/sra/SRX7293032

## Value of the Data

• The draft genomes data of *Bacillus* sp. strain MCMY6 and MCMY13 and *Pseudomonas* sp. MCMY3 and MCMY15 will provide the information of its ecology and genetic to enable further scientific investigations and comparisons.
• These draft genomes data are useful for the scientific communities that are working with the environmental bioremediation and toxicity studies of this contaminated site.
• Draft genome data drives the acceleration of functional genetic studies.

## 1 Data Description

**Fig. 1:**
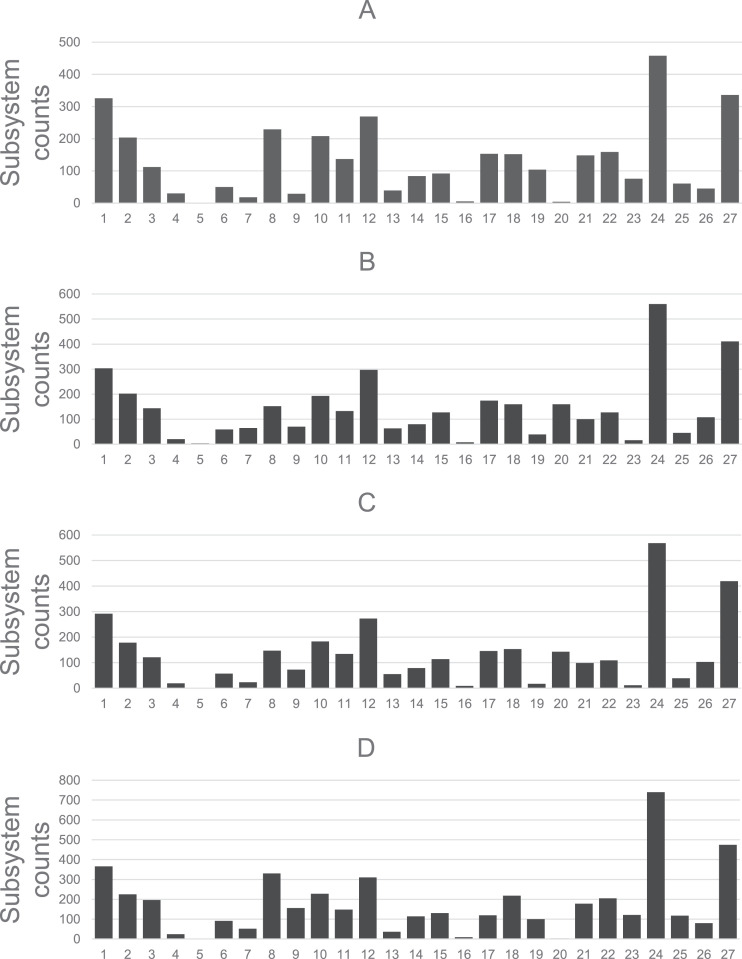

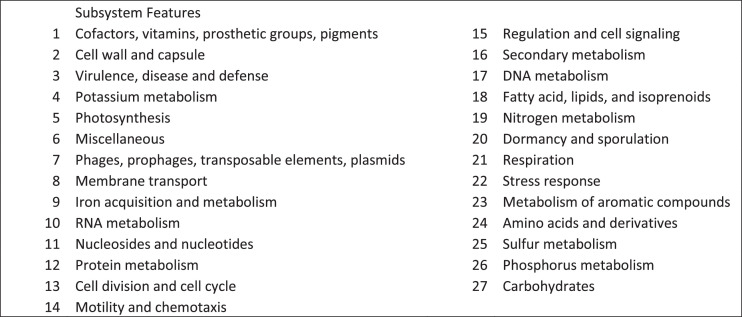
Overview of the subsystem categories annotated to (A) MCMY3, (B) MCMY6, (C) MCMY13, and (D) MCMY15 generated by RAST server.

Four draft genomes sequenced from soil bacteria strains from the abandoned Mamut Copper Mine, an anthropogenically polluted site that has been documented and classified as an AMD site [**1**]. Strains MCMY3 and MCMY15 were identified to be *Pseudomonas* spp. and strains MCMY6 and MCMY13 were identified to be closely related with the *Bacillus cereus* sensu lato lineage. All four genomes were sequenced using Illumina Hiseq platform with the paired-end format. The genome of *Pseudomonas* sp. MCMY15 showed the greatest genome length among the four strains with 7,381,777 bp and 64.8% of GC content, followed by the *Bacillus* sp. MCMY6 with 6,815,573 bp and 35.2% of GC content, *Pseudomonas* sp. MCMY3 with 6,396,595 bp and a GC content of 63.3% and lastly, *Bacillus* sp. strain MCMY13 with 5,559,059 bp and 35.5% of GC content. The annotation reports generated by RAST server [**2**] indicated that the genome of *Pseudomonas* sp. strain MCMY3 was estimated to have 4741 coding sequences classified in 493 subsystems and 76 RNA (Fig. 1**(A)**). The genome of *Bacillus* sp. MCMY6 contained 6865 protein-coding sequences, 89 RNA and they were organized in 495 subsystems (Fig. 1**(B)**). The genome of *Bacillus* sp. MCMY13 contained 6582 coding sequences in 495 subsystems, 70 RNA sequences (Fig. 1**(C)**). The genome of *Pseudomonas* sp. strain MCMY15 harbored 6582 protein coding sequences, 81 RNA genes and these sequences were classified into 579 subsystems (Fig. 1**(D)**).

**Fig. 2:**
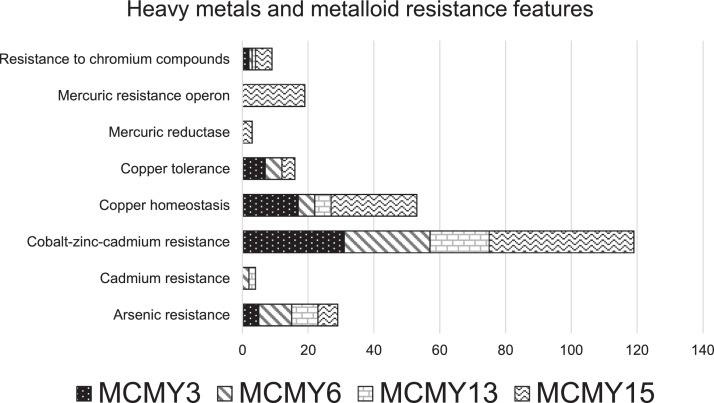
Heavy metals and metalloids resistance gene features detected in the four genomes.

The four bacterial strains contained, various heavy metals and metalloid resistance features which were observed in their genomes (Fig. 2). All four strains were found to be endowed with subsystems that were related with the resistance of chromium (Cr), copper (Cu), cobalt (Co), zinc (Zn), cadmium (Cd) and arsenic (As). The metal resistance gene features with the greatest abundance were observed among the four strains belongs to the subsystem of cobalt-zinc-cadmium resistance. This subsystem encodes the CzcABC protein complex that confers resistance to Co^2+^, Zn^2+^ and Cd^2+^ ions through cation efflux system [**3****,****4**] Furthermore, gene features for copper homeostasis and tolerance subsystems were found in all four bacterial genomes. Genes belongs to the copper resistance system (CopABCDZ) and their operon repressor (CsoR) were found to be present in all four strains, these genes are encode the copper binding and sequestration proteins that control the concentrations of Cu^2+^ ions within the intercellular medium [**5**], while other copper tolerance feature such as the copper homeostasis protein (CutE) and membrane protein for copper uptake (YcnI) were also detected. A total of 29 subsystem features pertained to arsenic resistance observed in the four genomes, including the arsenic resistance protein ArsH and ACR3, the operon repressor, arsenic pump-driving ATPase, arsenic efflux pump protein and arsenate reductase. In addition, the genome of *Pseudomonas* sp. MCMY15 contained a number of mercuric resistance features, such as the mercuric ion reductase, mercuric resistance operon coregulator, mercuric transport protein and mercuric resistance operon regulatory protein.

## 2 Experimental Design, Materials and Methods

All four bacteria strains were isolated from the soil sample collected from the former Mamut Copper Mine AMD site. The soil sample where strain MCMY3, 13 and 15 were isolated from the location 6.030324°; 116.657833° and strain MCMY13 was isolated from the soil collected from the location 6.030845°; 116.661892°. The method of isolation was performed as per the protocols stated by Low et al. [**6**]. The total gDNA samples of all strains were extracted and purified using QIAGEN DNeasy Blood and Tissue kits as per the manufacturer protocol. The quality and quantity of the gDNA samples extracted were assessed using agarose gel electrophoresis and Nanodrop 2000 spectrophotometer (Thermo Fisher Scientific, USA). The genomic DNA were sequenced by Novogene Co., Ltd., library preparation was done using the NEB Next Ultra DNA library Kit (Illumina) and the genomes was sequenced using the Illumina Hiseq 1500 platform with paired-end format. The sequences were trimmed by using Trimmomatic version 0.38 and assembled with the assistance of the SPAdes assembler version 3.11. The sequences were then uploaded to Rapid Annotation using Subsystem Technology (RAST) for annotation.

## Declaration of Competing Interest

The authors declare that they have no known competing financial interests or personal relationships which have, or could be perceived to have, influenced the work reported in this article.

## Appendix Supplementary Materials

Image, application 1
